# Supplementary material for: Pyruvate Dehydrogenase Kinase 1 inhibition mediated oxidative phosphorylation enhancement in cartilage promotes osteoarthritis progression
Source: BMC Musculoskelet Disord. 2023 Jul 20;24:597. doi: 10.1186/s12891-023-06585-6 (PMC10357736; doi:10.1186/s12891-023-06585-6)
Supplement: Supplementary file 1 — Additional file 1. [file 12891_2023_6585_MOESM1_ESM.zip › MF.Gsea.1653627316242/GOMF_DISULFIDE_OXIDOREDUCTASE_ACTIVITY.html]

Details for gene set GOMF\_DISULFIDE\_OXIDOREDUCTASE\_ACTIVITY[GSEA]

|  || Dataset | 55457\_collapsed\_to\_symbols.cls.cls #OA\_versus\_normal.cls.cls #OA\_versus\_normal\_repos |
| Phenotype | cls.cls#OA\_versus\_normal\_repos |
| Upregulated in class | OA |
| GeneSet | GOMF\_DISULFIDE\_OXIDOREDUCTASE\_ACTIVITY |
| Enrichment Score (ES) | 0.42704898 |
| Normalized Enrichment Score (NES) | 1.5861143 |
| Nominal p-value | 0.014742015 |
| FDR q-value | 0.14429124 |
| FWER p-Value | 0.989 |
Table: GSEA Results Summary

  

Fig 1: Enrichment plot: GOMF\_DISULFIDE\_OXIDOREDUCTASE\_ACTIVITY      
 Profile of the Running ES Score & Positions of GeneSet Members on the Rank Ordered List

  

| SYMBOL | TITLE | RANK IN GENE LIST | RANK METRIC SCORE | RUNNING ES | CORE ENRICHMENT || 1 | GLRX5 | glutaredoxin 5 [Source:HGNC Symbol;Acc:HGNC:20134] | 263 | 0.778 | 0.0667 | Yes |
| 2 | PDIA5 | protein disulfide isomerase family A member 5 [Source:HGNC Symbol;Acc:HGNC:24811] | 324 | 0.735 | 0.1443 | Yes |
| 3 | DNAJC10 | DnaJ heat shock protein family (Hsp40) member C10 [Source:HGNC Symbol;Acc:HGNC:24637] | 410 | 0.702 | 0.2163 | Yes |
| 4 | ENOX1 | ecto-NOX disulfide-thiol exchanger 1 [Source:HGNC Symbol;Acc:HGNC:25474] | 558 | 0.638 | 0.2763 | Yes |
| 5 | SELENOT | selenoprotein T [Source:HGNC Symbol;Acc:HGNC:18136] | 639 | 0.609 | 0.3383 | Yes |
| 6 | P4HB | prolyl 4-hydroxylase subunit beta [Source:HGNC Symbol;Acc:HGNC:8548] | 777 | 0.575 | 0.3920 | Yes |
| 7 | GFER | "growth factor, augmenter of liver regeneration [Source:HGNC Symbol;Acc:HGNC:4236]" | 1075 | 0.500 | 0.4250 | Yes |
| 8 | TXNRD3 | thioredoxin reductase 3 [Source:HGNC Symbol;Acc:HGNC:20667] | 2049 | 0.356 | 0.3893 | Yes |
| 9 | PDIA6 | protein disulfide isomerase family A member 6 [Source:HGNC Symbol;Acc:HGNC:30168] | 2073 | 0.353 | 0.4270 | Yes |
| 10 | CCS | copper chaperone for superoxide dismutase [Source:HGNC Symbol;Acc:HGNC:1613] | 2727 | 0.278 | 0.4074 | No |
| 11 | GLRX3 | glutaredoxin 3 [Source:HGNC Symbol;Acc:HGNC:15987] | 3336 | 0.218 | 0.3845 | No |
| 12 | ERO1A | endoplasmic reticulum oxidoreductase 1 alpha [Source:HGNC Symbol;Acc:HGNC:13280] | 3686 | 0.187 | 0.3783 | No |
| 13 | PDIA4 | protein disulfide isomerase family A member 4 [Source:HGNC Symbol;Acc:HGNC:30167] | 3756 | 0.181 | 0.3932 | No |
| 14 | ENOX2 | ecto-NOX disulfide-thiol exchanger 2 [Source:HGNC Symbol;Acc:HGNC:2259] | 3769 | 0.179 | 0.4124 | No |
| 15 | TXNL1 | thioredoxin like 1 [Source:HGNC Symbol;Acc:HGNC:12436] | 4135 | 0.143 | 0.4000 | No |
| 16 | TMX2 | thioredoxin related transmembrane protein 2 [Source:HGNC Symbol;Acc:HGNC:30739] | 4245 | 0.133 | 0.4065 | No |
| 17 | GSR | glutathione-disulfide reductase [Source:HGNC Symbol;Acc:HGNC:4623] | 4597 | 0.105 | 0.3909 | No |
| 18 | TMX1 | thioredoxin related transmembrane protein 1 [Source:HGNC Symbol;Acc:HGNC:15487] | 5101 | 0.064 | 0.3590 | No |
| 19 | STAB1 | stabilin 1 [Source:HGNC Symbol;Acc:HGNC:18628] | 5966 | -0.002 | 0.2921 | No |
| 20 | TXN2 | thioredoxin 2 [Source:HGNC Symbol;Acc:HGNC:17772] | 6072 | -0.010 | 0.2851 | No |
| 21 | GLRX2 | glutaredoxin 2 [Source:HGNC Symbol;Acc:HGNC:16065] | 6116 | -0.014 | 0.2833 | No |
| 22 | QSOX1 | quiescin sulfhydryl oxidase 1 [Source:HGNC Symbol;Acc:HGNC:9756] | 6130 | -0.015 | 0.2840 | No |
| 23 | GSTO1 | glutathione S-transferase omega 1 [Source:HGNC Symbol;Acc:HGNC:13312] | 6275 | -0.025 | 0.2756 | No |
| 24 | PDIA3 | protein disulfide isomerase family A member 3 [Source:HGNC Symbol;Acc:HGNC:4606] | 6607 | -0.050 | 0.2555 | No |
| 25 | TXNRD1 | thioredoxin reductase 1 [Source:HGNC Symbol;Acc:HGNC:12437] | 6820 | -0.064 | 0.2462 | No |
| 26 | NXN | nucleoredoxin [Source:HGNC Symbol;Acc:HGNC:18008] | 7376 | -0.108 | 0.2152 | No |
| 27 | GLRX | glutaredoxin [Source:HGNC Symbol;Acc:HGNC:4330] | 7554 | -0.123 | 0.2153 | No |
| 28 | STAB2 | stabilin 2 [Source:HGNC Symbol;Acc:HGNC:18629] | 7680 | -0.133 | 0.2205 | No |
| 29 | ERO1B | endoplasmic reticulum oxidoreductase 1 beta [Source:HGNC Symbol;Acc:HGNC:14355] | 7954 | -0.152 | 0.2162 | No |
| 30 | PGK1 | phosphoglycerate kinase 1 [Source:HGNC Symbol;Acc:HGNC:8896] | 8513 | -0.195 | 0.1946 | No |
| 31 | PDIA2 | protein disulfide isomerase family A member 2 [Source:HGNC Symbol;Acc:HGNC:14180] | 8961 | -0.229 | 0.1855 | No |
| 32 | TXN | thioredoxin [Source:HGNC Symbol;Acc:HGNC:12435] | 9487 | -0.273 | 0.1753 | No |
| 33 | TXNRD2 | thioredoxin reductase 2 [Source:HGNC Symbol;Acc:HGNC:18155] | 9922 | -0.314 | 0.1767 | No |
| 34 | SCO2 | synthesis of cytochrome C oxidase 2 [Source:HGNC Symbol;Acc:HGNC:10604] | 11410 | -0.488 | 0.1158 | No |
Table: GSEA details [plain text format]

  

Fig 2: GOMF\_DISULFIDE\_OXIDOREDUCTASE\_ACTIVITY      
 Blue-Pink O' Gram in the Space of the Analyzed GeneSet

  

Fig 3: GOMF\_DISULFIDE\_OXIDOREDUCTASE\_ACTIVITY: Random ES distribution      
 Gene set null distribution of ES for **GOMF\_DISULFIDE\_OXIDOREDUCTASE\_ACTIVITY**
